# Identifying and Ranking Strategies to Address Housing Insecurity and Homelessness Within the LGBTQIA+ Community in Southern Nevada: Utilization of Community-Based Participatory Research and Concept Mapping

**DOI:** 10.3390/ijerph21121540

**Published:** 2024-11-21

**Authors:** Emylia Terry, Jennifer Pharr, Renato M. Liboro, Courtney Coughenour, Krystal Kittle, John Waldron, Jason D. Flatt

**Affiliations:** 1 Department of Environmental and Occupational Health, School of Public Health, University of Nevada, Las Vegas, Las Vegas, NV 89154, USA; jennifer.pharr@unlv.edu (J.P.); courtney.coughenour@unlv.edu (C.C.); 2 Department of Psychology, College of Liberal Arts, University of Nevada, Las Vegas, Las Vegas, NV 89154, USA; renato.liboro@unlv.edu; 3 Department of Community Health Education, School of Public Health and Health Sciences, University of Massachusetts Amherst, Amherst, MA 01003, USA; kkittle@umass.edu; 4 The LGBTQIA+ Community Center of Southern Nevada, Las Vegas, NV 89101, USA; jwaldron@thecenterlv.org; 5 Department of Social and Behavioral Health, School of Public Health, University of Nevada, Las Vegas, Las Vegas, NV 89154, USA; jason.flatt@unlv.edu

**Keywords:** housing insecurity, LGBTQIA+, community-based participatory research, concept mapping

## Abstract

Housing insecurity is a critical issue within Southern Nevada. However, little is known about the housing-insecurity-related needs of LGBTQIA+ Southern Nevadans. The aim of this study was to identify strategies to address housing insecurity among this vulnerable community. Utilizing community-based participatory research and concept mapping, the most salient solutions were identified and prioritized at a Community Housing Forum. This Forum brought together stakeholders with expertise in housing or who work with the LGBTQIA+ community. The most important identified solutions consistently emphasized the criticality of culturally competent mental health services; the need for affordable housing options; and various social and environmental factors. There is a continued need for research and collaboration among organizations and providers to better serve LGBTQIA+ individuals experiencing housing insecurity. Additional research is needed to determine the efficacy of the identified solutions and to inform the development of context-specific and broadly applicable strategies to address housing insecurity within this community.

## 1. Introduction

Housing insecurity is a term that encapsulates various aspects of housing problems that people may experience, such as safety, affordability, and quality [1]. Housing insecurity is a critical social determinant of health, impacting health outcomes, health risks, and overall well-being [2]. Housing insecurity includes various challenges, such as difficulties affording rent, and can negatively impact healthcare access and physical health [3,4,5]. Cost-burdened households are those that spend more than 30% of income on housing costs, leaving little left for necessities including healthcare, clothing, and food [4,6,7]. Notably, within the United States (U.S.), Black and Hispanic households are nearly twice as likely to be cost-burdened compared to white households [8]. Individuals facing housing instability are at risk of experiencing homelessness, which represents the most severe form of housing deprivation [9].

According to various studies, individuals experiencing homelessness have higher rates of chronic diseases such as diabetes and substance misuse, face nearly twice the risk of premature death, and have a mortality rate of nine to ten times higher than the general population [10,11]. While 11 out of every 10,000 people experiencing homelessness are White, 48 out of every 10,000 are Black, and 121 out of every 10,000 are Native Hawaiian or Pacific Islanders; these statistics reflect the systemic oppression that many groups face, such as exclusionary housing policies and displacement [12].

The literature is limited with regard to individuals who identify as lesbian, gay, bisexual, transgender, queer, intersex, and asexual, as well as additional identities (LGBTQIA+), experiencing housing insecurity and homelessness. According to the UCLA Williams Institute, LGBTQIA+ people experience higher rates of poverty, housing instability, and homelessness compared to cisgender (not transgender), heterosexual individuals [13]. For example, almost 22% of LGBTQIA+ adults in the U.S. experience poverty, versus 15.7% of non-LGBTQIA+ adults [13]. Further, within the LGBTQIA+ community, poverty is particularly prevalent among racial- and ethnic-minority individuals, women, transgender people, bisexual people, and younger individuals [13]. Among homeless youth, between 20% to 45% identify as being part of the LGBTQIA+ community, despite comprising only 9.5% of the U.S. population aged 13 to 17 [13,14]. Moreover, LGBTQIA+ people report facing extensive discrimination when trying to access housing-related services [13].

The COVID-19 pandemic greatly impacted residents in Southern Nevada, increasing housing insecurity by 12.58 percentage points between December 2020 and December 2021, and Nevadans of color faced disproportionate rates of housing insecurity compared with White Nevadans [15]. Further, Nevadans disproportionately experience homelessness, with 24.2 of every 10,000 people experiencing homelessness in 2022, compared to the national average of 18 per 10,000 [16]. Much of this issue exists within southern Nevada, as Las Vegas is the largest metropolitan area in the state and is ranked within the U.S.’s top ten cities with the highest rates of homelessness [17]. The situation has continued to worsen, with a 14% increase in homelessness in Las Vegas between 2022 to 2023 [18]. LGBTQIA+ Southern Nevadans may be particularly vulnerable.

It is estimated that 5.5% of Nevadans identify as part of the LGBTQIA+ community, with 52% being from racial- or ethnic-minority backgrounds, making Nevada the third highest LGBTQIA+-populated state in the country [19]. However, very little is known about the housing needs of LGBTQIA+ people in Southern Nevada. “Building Healthy Outcomes Using a Supportive Environment (H.O.U.S.E.) Southern Nevada” is an interdisciplinary, community-based participatory research (CBPR) project with the goal of examining and developing supportive housing options for LGBTQIA+ people in Southern Nevada.

Our Building H.O.U.S.E. team conducted a 2021–2022 community needs assessment that obtained responses from 375 LGBTQIA+ Southern Nevadans and found that 22% reported a household income of less than $10,000 [20]. This underscores the importance of Building H.O.U.S.E., which unites relevant community stakeholders, key opinion leaders, and organizations together to identify strategies to understand and address the housing and health needs of diverse LGBTQIA+ Southern Nevadans.

The purpose of this study was to identify ways to address housing insecurity and homelessness among LGBTQIA+ Southern Nevadans. In partnership with key community representatives and leaders with the relevant expertise to generate potential strategies to mitigate housing insecurity and homelessness, our team utilized concept mapping to identify and prioritize the most salient solutions.

## 2. Methods

### 2.1. Study Population

CBPR in public health represents an active partnership between community members, key stakeholders, and researchers throughout all phases of research [21]. Each partner shares expertise in actions aimed at benefitting the community involved [21]. In line with CBPR tenets and practices such as collaboration; capacity-building; the meaningful involvement of relevant stakeholders in the research process; and shared governance, responsibility, and ownership, our team worked together with our Southern Nevada community partners to establish a Building H.O.U.S.E. Advisory Board [22,23,24,25]. We recruited members for our board based on their expertise working with the LGBTQIA+ population and/or addressing housing insecurity or homelessness within Southern Nevada, with meetings taking place virtually on a quarterly basis. In March 2022, our team conducted key informant interviews with 12 stakeholders from Southern Nevada to gain insight into the health, social, and environmental factors specific to our region and identify potential leaders with knowledge of housing insecurity and homelessness. Participants were from LGBTQIA+-related community organizations or local housing/homelessness service providers. After reviewing our interview transcripts, we identified unique social determinants of health including discrimination, food insecurity, and limited resources that likely contribute to housing insecurity and homelessness. Informants also raised concerns around affordable housing, housing availability, and LGBTQIA+-friendly housing, with one pointing out common community stressors such as disownment and trauma. Within this context, we held a Community Housing Forum utilizing concept mapping (CM) in person in April 2022 at The LGBTQIA+ Community Center of Southern Nevada. Participants included those with expertise in housing-related services; they held a range of roles in community-based not-for-profits, such as case workers, peer advocates, and program managers. Participants were recruited by leveraging our connections with the Advisory Board, who reached out to their colleagues and networks.

### 2.2. Study Design

We utilized the Concept System^®^ Global MAX™ and GroupWisdom™ web platform to capture suggested solutions to address homelessness and housing insecurity within Southern Nevada’s LGBTQIA+ community and develop a concept map during the Community Housing Forum. These pieces of software are unique in that they allow for the organization and visualization of complex ideas to foster efficient and collaborative decision-making and outcomes [26]. CM, with its emphasis on participatory processes, has been used in past CBPR work to brainstorm, thematically cluster, and rate strategies to engage and recruit LGBTQIA+ participants in Alzheimer's disease and related dementias’ research [27]. CM’s participatory, mixed-methods approach was particularly relevant to this work, as it involves collecting qualitative data prior to quantitative data through an exploratory sequential process [28,29,30]. The three phases utilized for the CM process included brainstorming within groups, clustering relevant solutions generated by each group (i.e., housing needs), and rating these emergent solutions by level of importance. We collected data in April 2022, with all research activities approved by the UNLV Institutional Review Board, Protocol # UNLV-2021-267.

### 2.3. Measures

In addition to CM participation, participants were also asked to provide demographic and employment data. Participants were asked the following questions: “Which of the following represents the primary focus of or service your organization provides?” (response options: housing services, health/healthcare services, advocacy/legal services, educational/research services, financial support services, social services, or other); “How many years have you been at your current organization?” (response option: open-ended); “Which of the following terms best describes your current gender identity? (check all that apply)” (response options: man, woman, transgender man or trans man, transgender woman or trans woman, non-binary or genderqueer, or another identity); “Which of the following terms best describes your sexual orientation? (check all that apply)” (response options: asexual, bisexual, gay or lesbian, heterosexual or straight, queer, or another sexual orientation); and “Have you ever experienced housing insecurity or been homeless?” (response options: yes, no).

During the brainstorming phase, participants broke out into ten different, nonrandom groups consisting of approximately four to five people, including graduate students and faculty members who served as facilitators for each group. Groups were asked, “Please list as many potential solutions as you can think of that may help to address housing insecurity or homelessness of the LGBTQIA+ community in Southern Nevada”. Groups were provided instructions and encouraged to discuss and provide responses as a group. Responses were then recorded by each of the facilitators from the ten groups in the GroupWisdom software using a laptop provided to each group, and groups were able to generate more than one potential strategy. After solutions were identified, the moderators cleaned the solutions and deleted duplicates, edited for grammar, and broke statements with multiple solutions into separate ideas.

**Figure 1:**
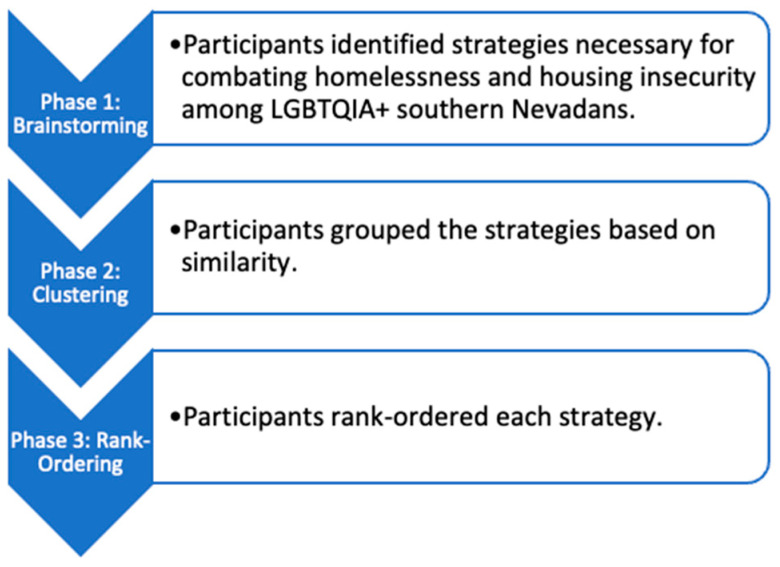
Concept mapping process.

During the clustering phase, each of the ten groups classified the cleaned solutions based on their similarities and named the strategies based on shared contents. Then, participants were asked to rate each strategy by importance from one to four (1 = relatively unimportant, 2 = slightly important, 3 = moderately important, and 4 = extremely important; Figure 1).

### 2.4. Statistical Analysis

We used the GroupWisdom™ web platform to conduct data analysis for the brainstorming, clustering, and rating phases [26]. First, based on the rated data, the software created a similarity matrix. Then, a non-metric, multidimensional scaling analysis of this matrix was employed to depict the arrangement of each solution on a two-dimensional (X, Y) “point map” [26,28]. We used cluster analysis available through the software to categorize the list of ideas into groups of related statements known as “cluster maps”, which were used to create the final concept map.

We used the software to calculate the mean level of importance assigned to each solution. We then studied the cluster maps to determine the optimal number of clusters that best represented the main thematic areas. Our team conducted an iterative process of review and discussion with the participants to generate a final concept map that demonstrated the most salient strategies, organized logically to align with and reflect participants’ expertise. This mixed-methods approach allowed for our team to quickly synthesize ideas from a vast group of service providers to determine the most critical needs and solutions to best address homelessness and housing insecurity among Southern Nevada’s LGBTQIA+ community.

## 3. Results

### 3.1. Participant Characteristics

A total of 40 people participated in the Community Housing Forum and CM process (Table 1). Most participants identified as women (55%). One in four (25%) identified as a gender minority (transgender, non-binary, or gender diverse), and more than half (55%) identified as LGBTQIA+. Participants worked in a variety of fields relevant to housing and homelessness, ranging from health and healthcare services (20%), housing services (17.5%), advocacy and legal services (5%), and social services (2.5%), with the largest groups working within educational/research services (30%) and other areas (25%). Over a third (42%) of participants reported that they had experienced housing insecurity or homelessness in the past. The duration of employment within their current organization ranged from 3 months to 22 years, with an average of 4.77 years (SD = 4.91).

### 3.2. Phase 1: Brainstorming

Ten groups were given the instruction to spend ten minutes brainstorming needs to address homelessness and housing insecurity among LGBTQIA+ Southern Nevadans. Each group wrote down their ideas within the GroupWisdom™ platform. At the end of the brainstorming phase, the data were cleaned (de-duplicated and edited) by facilitators. A list of 124 statements were presented for final review and CM using GroupWisdom™ (Table S1).

### 3.3. Phase 2: Clustering

**Figure 2:**
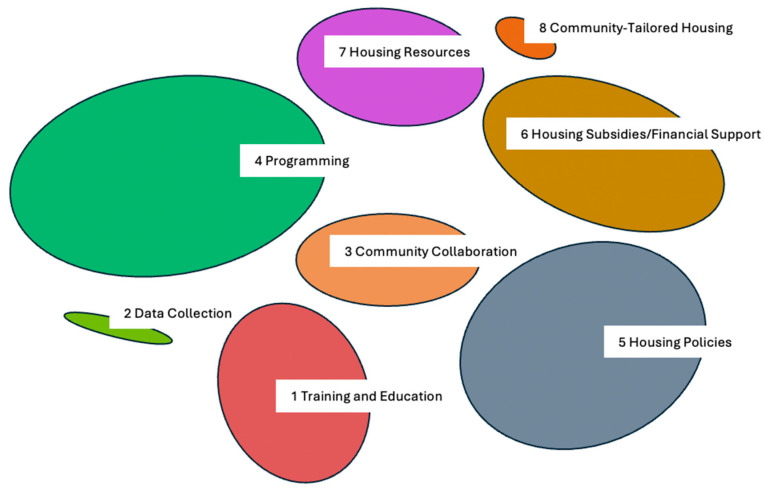
Clusters obtained from CM activity according to Community Housing Forum participants in Southern Nevada in April 2022 (N = 40).

The 124 statements were then clustered by similarity by each group using the GroupWisdom™ platform. The following clusters emerged as important areas for focus and intervention: education and training, data collection, community collaboration, programming, housing policies, housing subsidies and financial support, housing resources, and community-tailored housing (Figure 2). The cluster sizes depict the overall relationship between solutions and ratings, with smaller clusters indicating solutions of lower importance.

### 3.4. Phase 3: Rating of Overall Importance of Solutions Within Each Cluster

As displayed in Table 2, the overall highest rated solution fell under the Community Collaboration cluster, collaboration between service providers (mean = 3.88). The most popular solution under the Community-Tailored Housing cluster was affordable, safe housing (mean = 3.71). The most popular solution falling under the Data Collection cluster was provide training for agencies/providers on working with and serving the LGBTQIA+ community (mean = 3.54). The most popular solution falling under the Housing Policies cluster was having strong advocates in government to protect LGBTQIA+ rights (mean = 3.79). The most popular solution falling under the Housing Resources cluster was the need for more safe housing options for trans people (mean = 3.75). The most popular solution falling under the Housing Subsidies/Financial Support was housing assistance for older LGBTQIA+ people (mean = 3.38). The most popular solution falling under the Programming cluster was friendly and accessible mental health services (mean = 3.67). Lastly, the most popular solution falling under the Training and Education cluster was the need for people from the LGBTQIA+ community to be intentionally involved in the development, implementation, and evaluation of services (mean = 3.71).

### 3.5. Phase 3: Overall Importance of Solutions

Mean scores represented the average ratings for each solution, rated from 1 (relatively unimportant) to 4 (extremely important). As demonstrated in Table 3, the top overall solutions by importance included the following: collaboration between service providers (mean = 3.88); strong advocates in government to protect LGBTQIA+ rights (mean = 3.79); more safe housing options for trans people (mean = 3.75); including LGBTQIA+ community members in every stage of developing, implementing, and evaluating services (mean = 3.71); collaboration between housing-related organizations (mean = 3.71); affordable, safe housing (mean = 3.71); the better enforcement of non-discrimination laws (mean = 3.67); the need for senior-specific LGBTQIA+ housing (mean = 3.67); connective/wraparound services (mean = 3.67); and friendly and accessible mental health services (mean = 3.67). All results were then shared back with participants at the Forum and in subsequent Advisory Board meetings, where members of the board were able to provide guidance on the conduct of the study and timely feedback on the findings. The Advisory Board members also participated in the Forum and CM. Finally, we also shared our results back to participants in a follow-up Community Forum in March of 2024.

## 4. Discussion

Employing CM with service providers in Southern Nevada through the utilization of software presented a novel, innovative approach to CBPR. This strategy, allowing for real-time cluster analysis with participants, yielded invaluable insights about what solutions and resources are needed to combat housing insecurity and homelessness within Southern Nevada’s LGBTQIA+ community. The following clusters emerged as critical areas for focus: education and training, data collection, community collaboration, programming, housing policies, housing subsidies and financial support, housing resources, and community-tailored housing. Within the housing policies cluster, two top-ranked solutions included the need to elect and maintain advocates in government to protect LGBTIQA+ rights and to have better enforcement of non-discrimination laws.

The literature supports the importance of these solutions. For example, LGBTQIA+ individuals experience housing discrimination, which exacerbates housing instability within this population [31,32]. LGBTQIA+ people also face challenges navigating homeless shelters and services, including violence, harassment, lack of cultural competency among staff, and anti-transgender policies [33]. The election and appointment of lawmakers who continue to fight for LGBTQIA+ Southern Nevadans, as well as the adoption and enforcement of comprehensive protections at the state and federal levels, may assist with mitigating the ongoing housing discrimination and resultant housing insecurity that this community continues to experience [33].

Further, it is well-established in the literature that family rejection is a common cause of LGBTQIA+ homelessness [34,35]. This underscores the importance of ideas that emerged within the community-tailored housing cluster. For example, to escape from intolerant households, young people may move to large urban cities to find community with other LGBTQIA+ individuals who also do not have stable housing [36]. However, housing insecurity and homelessness are not limited to LGBTQIA+ youth. LGBTQIA+ older adults may be particularly vulnerable to housing insecurity due to economic vulnerability and discrimination. For example, research found that different-sex older couples approaching retirement had an income that was 4.3 times higher than same-sex older couples, and a majority of LGBTQIA+ AARP survey respondents aged 45+ reported concerns about identity-based neglect, service refusal, and harassment in long-term care facilities [37,38].

Additionally, due in large part to stigma and discrimination, 30% of transgender people reported experiencing homelessness at some point in their lives, and only 25% of transgender adults were homeowners in 2015, compared to 58% of cisgender adults [33,39,40]. When transgender people seek assistance from shelters, they are often housed in sex-segregated facilities in accordance with the sex they were assigned at birth, leading many to remain unsheltered [33]. This highlights the need for housing that is culturally competent and inclusive for LGBTQIA+ community members. For example, a recent study focused on the experiences of community members who moved into senior LGBTQIA+ housing. Participants reported benefits associated with the housing, such as better physical health, psychological health, and overall well-being [41]. Thus, the importance of the community-tailored housing cluster cannot be understated.

Poverty is the instrumental driver of homelessness, and homelessness, in turn, makes escaping poverty more difficult, underscoring the importance of the housing subsidies and financial support cluster [42,43,44]. Notably, poverty can also lead to addiction and poor mental health, directly affecting how individuals experience homelessness [44,45]. For example, there is a bidirectional relationship between mental health and homelessness: experiencing homelessness can reduce mental well-being, and mental illness can lead to experiencing homelessness [46]. Further, 42–80% of people experiencing homelessness report mental health struggles, while 40% of non-homeless LGBTQIA+ individuals report the same [47,48,49]. Thus, poor mental health is a considerable issue among those experiencing homelessness, LGBTQIA+ people in general, and homeless LGBTQIA+ people, highlighting the need for affirming mental health professionals and the benefit of housing with wraparound services such as employment, financial literacy, and addiction resources [45]. Housing with wraparound services (such as financial literacy, healthcare, and employment resources) and increased access to culturally competent mental health professionals thus emerged as important solutions falling under the housing resources and programming clusters, respectively.

The above highlights the importance of some of the highest-ranked solutions identified by service providers through CM: the need for LGBTQIA+ advocates in government and the enforcement of anti-discrimination laws and policies, the need for safe and affordable housing, the creation of housing options for LGBTQIA+ older adults and transgender people, and the design of housing with wraparound services, such as employment and healthcare resources. Because experiences with homelessness and housing insecurity interact “in complex ways with sexual and gender identity… tailored mainstream housing provision is required for LGBT[QIA]+ homeless people;” in other words, to effectively mitigate LGBTQIA+ housing insecurity, housing options must be personalized to the various needs of this diverse community [50]. In short, it is vital to expand shelter and housing options for LGBTQIA+ individuals so that they are safely and appropriately sheltered [33]. This stresses the need for cultural competency trainings for staff across all agencies that interface with LGBTQIA+ populations so that members of this community are supportively and affirmingly housed while having mental health, healthcare, and other needs satisfactorily met [33].

To our knowledge, this study is the first of its kind that compiles solutions to address homelessness and housing insecurity among LGBTQIA+ Southern Nevadans. Despite its valuable contributions, it is not without limitations. One such limitation is its cross-sectional design, which may have captured strategies that may not always be relevant or applicable. Additionally, while the involvement of service providers provided important perspectives, we may have missed those with additional valuable insights, including those actively experiencing homelessness or housing insecurity. Further, participants were recruited based on convenience sampling and were not randomly sorted into the ten groups, and we may not have captured all the details in the discussions and potential disagreements from each group. Lastly, these findings may not be generalizable outside of Southern Nevada. Despite these drawbacks, this research is pivotal to the next phase of our praxis, which involves soliciting feedback on the most salient CM strategies from LGBTQIA+ Southern Nevadans experiencing homelessness or housing insecurity. This feedback will, in turn, inform our team’s implementation efforts, wherein the most relevant solutions will be deployed in partnership with the community to help alleviate homelessness and housing insecurity within our community.

## 5. Conclusions

The solutions discussed within this paper offer valuable insights and actionable recommendations for researchers, institutions, community-based organizations, and policymakers seeking to combat homelessness and housing insecurity among LGBTQIA+ individuals in urban areas. The suggestions generated by relevant service providers consistently emphasized the importance of housing options that are tailored to the unique needs of this diverse community, with a focus on cultural competency among mental health practitioners and service providers. This work emphasizes the continued need for collaboration and community among organizations, providers, and the communities they serve to better address homelessness and housing insecurity among LGBTQIA+ Southern Nevadans. Future research should expand on this knowledge by developing both broadly applicable and context-specific strategies for alleviating housing insecurity and homelessness among the LGBTQIA+ community.

## Figures and Tables

**Table 1:** Characteristics of Community Housing Forum participants from southern Nevada in April 2022 (N = 40).

Characteristic	Frequency	Percentage
Sexual orientation		
Asexual	2	5.26
Bisexual	5	13.16
Gay or Lesbian	10	26.32
Heterosexual or straight	18	47.37
Queer	5	13.16
Gender Identity		
Man	9	23.68
Woman	21	55.26
Transgender man or trans man	1	2.63
Transgender woman or trans woman	1	2.63
Non-binary or genderqueer	8	21.05
Which of the follow represents the primary focus of or service your organization provides?		
Housing services	7	17.5
Health/Healthcare services	8	20
Advocacy/Legal services	2	5
Educational/Research services	12	30
Financial support service	0	0
Social services	1	2.5
Other	10	25
Have you ever experienced housing insecurity or been homeless?		
Yes	16	42.11
No	22	57.89
How many years have you been at your current organization? (Mean, Standard Deviation)	4.77	4.91

**Table 2:** Most important solutions within each cluster according to Community Housing Forum participants in Southern Nevada in April 2022 (N = 40).

Solutions	Mean Score	Clusters
1. Affordable, safe housing	3.71	Community-Tailored Housing
2. Collaboration between service providers	3.88	Community Collaboration
3. Friendly and accessible mental health services	3.67	Programming
4. Housing assistance for older LGBTQIA+ people	3.38	Housing Subsidies/Financial Support
5. More safe housing options for trans people	3.75	Housing Resources
6. Provide training for agencies/providers on working with and serving the LGBTQIA+ community.	3.54	Data Collection
7. Strong advocates in government to protect LGBTQIA+ rights	3.79	Housing Policies
8. Whatever we do, people from the LGBTQIA+ community need to be intentionally involved in the development, implementation, and evaluation of services	3.71	Training and Education

**Table 3:** Most important solutions by mean score rating according to Community Housing Forum participants in Southern Nevada in April 2022 (N = 40).

Solutions	Mean Score	Cluster
1. Collaboration between service providers	3.88	Community Collaboration
2. Strong advocates in government to protect LGBTQIA+ rights	3.79	Housing Policies
3. More safe housing options for trans people	3.75	Housing Resources
4. Whatever we do, people from the LGBTQIA+ community need to be intentionally involved in the development, implementation, and evaluation of services	3.71	Training and Education
5. All groups dealing with housing need to work together	3.71	Community Collaboration
6. Affordable, safe housing	3.71	Community-Tailored Housing
7. Better policing of non-discrimination laws	3.67	Housing Policies
8. Senior LGBTQIA+ housing	3.67	Community-Tailored Housing
9. Connective/wraparound services	3.67	Housing Resources
10. Friendly and accessible mental health services	3.67	Programming

## Data Availability

The data presented in this study are available upon request from the corresponding author.

## Supplementary Materials

The following supporting information can be downloaded at: https://www.mdpi.com/article/10.3390/ijerph21121540/s1, Table S1: Statements generated by the Community Housing Forum participants during the brainstorming phase (N = 124).
