# Supplementary material for: Identifying and Ranking Strategies to Address Housing Insecurity and Homelessness Within the LGBTQIA+ Community in Southern Nevada: Utilization of Community-Based Participatory Research and Concept Mapping
Source: Int J Environ Res Public Health. 2024 Nov 21;21(12):1540. doi: 10.3390/ijerph21121540 (PMC11675821; doi:10.3390/ijerph21121540)
Supplement: Supplementary file 1 [file ijerph-21-01540-s001.zip › ijerph-3286450-supplementary.pdf]

Table S1. Statements generated by the Community Housing Forum participants during the brainstorming phase (N = 124).

Statements/Strategies		Bridging
	Cluster name: Family	Avg 0.33
1	A "know your rights" training for folx to be able to advocate for themselves around housing and other needs.	0.25
2	LGBTQ+ training for people who provide housing services	0.35
4	Easy access to information	0.32
7	Training for assisted living and LTC staff and residents	0.23
25	Trans people of color need to be centered in the work and solutions.	0.29
26	Encourage/require homeless management information systems (HMIS) to collect sexual orientation gender identity and expression (SOGIE) data	0.62
30	Whatever we do, people from the LGBTQIA+ need to be intentionally involved in the development, implementation and evaluation of services	0.36
40	Community Education	0.22
45	Employers that aren't discriminatory against trans and non-binary folx. There already exist anti-work discrimination laws but enforcement seems to be an issue.	0.34
49	Stigma reduction	0.22
58	Normalize LGBTQIA+ families	0.27
68	Inclusive and affirming landlords	0.27
69	Create reform by providing information to Landlords/Realtors/Businesses that rent to LGBTQIA+ community so they are aware of housing concerns and issues	0.2
72	Pre-existing housing providers build internal capacity, through trainings, inclusive policies and procedures; etc. to house more LGBTQ+ folks.	0.32
73	Family intervention to support prevention of youth homelessness when coming out	0.24
93	Collect more data on the LGBTQ+ community focused on housing needs.	0.74
	Family Cluster Statistics	
	Statement amount: 16	Average: 0.328
	Median: 0.283	Variance: 0.02
	Standard deviation: 0.142	Minimum: 0.204
	Maximum: 0.736	
	Cluster name: Data/Research	Avg 0.16
6	LGBTQ+ competency training for landlords and realtors	0.06
18	Find out the needs of populations that are underrepresented in the needs assessment	0.36
24	Do census of homeless vets to identify those who need housing.	0.36
43	Training specific to LGBTQ housing experiences at affordable housing agencies, realtor boards, and housing boards.	0.06
88	Provide training for agencies/providers on working with and serving the LGBTQ+ community.	0.06
100	Education	0.16

112	Resources and training.	0.06
	Data/Research Cluster Statistics	
	Statement amount: 7	Average: 0.162
	Median: 0.061	Variance: 0.017
	Standard deviation: 0.131	Minimum: 0.061
	Maximum: 0.362	
	Cluster name: Collaborations	Avg 0.42
3	Low barriers to accessible and low-income housing.	0.44
38	Easier access to case management for housing vouchers	0.41
39	Supporting individuals who have experienced trauma by providing access to services (i.e. sexual assault, domestic violence) that are inclusive & affirming.	0.44
71	All groups dealing with housing need to work together.	0.47
94	Meditation between tenant and landlords	0.41
107	support with legal housing issues	0.38
118	Collaboration between service providers	0.36
	Collaborations Cluster Statistics	
	Statement amount: 7	Average: 0.416
	Median: 0.412	Variance: 0.001
	Standard deviation: 0.035	Minimum: 0.357
	Maximum: 0.468	
	Cluster name: Programs	Avg 0.39
5	An information hub/website that gives information on LGBTQIA+ friendly housing	0.3
16	Supporting LGBTQIA youth	0.39
17	Increase access to career opportunities or trades with emphasis on the transgender community.	0.6
19	Safe and affirming residential mental health and substance abuse treatment facilities.	0.27
28	Good lighting	1
29	Trauma centric programming and case management to assist with addressing mental health, employment, and housing	0.26
34	LGBTQIA+ employment/vocational programs that place participants with LGBTQIA+ friendly employers for sustainability after they are housed	0.4
46	Improve shelter services to be better, overall, and more inclusive and supportive of transgender men and women.	0.47
50	Increase the number of housing providers that are willing and able to implement and manage LGBTQ+ tailored housing.	0.41

55	Offer LGBTQIA+ friendly and inviting financial guidance classes.	0.3
60	trauma counselors on site	0.32
65	Competent case management	0.25
70	Create educational job rehab programs	0.47
74	Friendly and accessible mental health services	0.27
78	mental health support	0.27
80	Benefit application assistance	0.27
92	Assistance to new residents	0.26
96	Housing counseling (help to maintain housing)	0.29
98	Housing navigation (help to locate housing)	0.25
102	Safe integration (LGBT and non-LGBT)	0.85
106	Teach the LGBTQIA+ community key concepts in navigating renting issues and concerns	0.3
114	Central hub for information and referral system	0.34
	Programs Cluster Statistics	
	Statement amount: 22	Average: 0.388
	Median: 0.302	Variance: 0.037
	Standard deviation: 0.193	Minimum: 0.249
	Maximum: 1	
	Cluster name: Legislation	Avg 0.16
8	better policing of non-discrimination laws	0.06
9	Strong advocates in government to protect LGBTQ rights	0.33
11	Tax the casino industry and work with legislators to improve housing funding.	0.04
13	Increase Clark County Fair Market Rent	0.28
14	Enforcement of fair housing laws. NV does not receive the full amount of federal monies available to enforce fair housing because we do not have state legislation that authorizes NV to establish a Fair Housing Assistance Program (FHAP).	0.04
31	Increase the number of Runaway and Homeless Youth Act (grant) providers i.e. street outreach, transitional living programs, basic center programs, which tend to have better LGBTQ+ inclusive policies woven into their grant opportunities & expectations	0.37
33	Expand source of income protections Statewide to stop discrimination against people using rent vouchers and rental assistance.	0.06
41	Implement Office of Human Rights at city levels that can do a better job of responding to human rights infractions, related to LGBTQ+ housing and homelessness,	0.04
47	Stop politicizing basic needs and human rights	0.27
52	Change zoning laws for high rise buildings for affordable housing.	0.01
53	democratize work	0.17
56	legalize sex work	0.04
57	Inspection/background process for prospective landlords	0.34
59	Limit land/real estate ownership by corporations	0.03
61	Limit land/real estate ownership by foreign individuals	0.07

64	Require resort/entertainment builders to allocate percentage of budget for building affordable housing.	0.08
77	Child Tax Credit	0.21
81	Employment assistance	0.41
82	More accessibility to Land	0.44
83	Decriminalization of sex work	0.04
89	Help LGBTQIA+ people remove misdemeanors from homeless citations to be able to qualify for housing	0.37
91	Universal basic income	0.15
95	Increased income supplementation	0.21
104	Inflation control	0.08
108	Expanding Medicaid to undocumented immigrants	0.24
111	higher minimum wage	0.05
113	Monitoring landlord activities	0.22
115	rent control	0.07
119	Livable wages	0.09
124	Rent control legislation	0.01
	Legislation Cluster Statistics	
	Statement amount: 30	Average: 0.16
	Median: 0.084	Variance: 0.018
	Standard deviation: 0.133	Minimum: 0.007
	Maximum: 0.444	
	Cluster name: Subsidies/Monetary Support	Avg 0.32
10	Provide separate transgender housing.	0.34
12	Transportation assistance	0.3
15	Higher housing vouchers as rent increases	0.29
20	Utility assistance	0.3
23	Rental and mortgage assistance with low barriers of approval	0.31
44	Long term shallow rent subsidies.	0.32
62	Gayborhoods that are affordable	0.29
66	Rent support	0.31
67	Housing grants to cover move in costs<br>Free Transitional housing for enough time to allow someone to get employed and start having regular income<br>Free/ low rent for trans youth (up to 24)	0.32
75	Fee waivers for applications (application fees, deposits, etc)	0.35
84	aging in place assistance	0.25
86	housing assistance for older LGBTQ+ people	0.28
87	Shared housing on separate leases	0.51
99	Housing subsidies	0.29
101	Short term rental assistance	0.29

105	Long term subsidy	0.32
123	Increasing Access to housing vouchers	0.34
	Subsidies/Monetary Support Cluster Statistics	
	Statement amount: 17	Average: 0.316
	Median: 0.309	Variance: 0.003
	Standard deviation: 0.054	Minimum: 0.246
	Maximum: 0.507	
	Cluster name: Homeless Shelter	Avg 0.29
21	We need a homeless shelter that serves the community then services and help to transition into housing	0.34
27	Security cameras	0.61
32	Case workers to help homeless LGBTQIA+ people find work, keep work, and progress in jobs	0.19
35	food pantries embedded in housing	0.24
36	Case workers to assist LGBTQIA+ individuals with mental health improvements	0.23
63	connective/wrap around services	0.19
76	Ongoing case management	0.19
97	More safe housing options for trans people	0.25
103	transitional housing	0.3
117	LGBTQ+ supportive housing community	0.52
121	Case management	0.19
122	More low-income housing opportunities	0.21
	Homeless Shelter Cluster Statistics	
	Statement amount: 12	Average: 0.289
	Median: 0.231	Variance: 0.018
	Standard deviation: 0.133	Minimum: 0.193
	Maximum: 0.613	
	Cluster name: Housing Types	Avg 0.04
22	Reserved housing (set asides)<br>Managed Care Organizations<br>Non-profit housing development<br>Covert empty office buildings Nd box stores into transitional dorms	0.04
37	Combined housing for LGBTQ+ youth and seniors.	0.01
42	Youth specific housing options	0.19
48	Shared housing models	0
51	senior LGBTQIA+ housing	0.04

54	Studio dorms for homeless LGBTQIA+ individuals	0.04
79	Considering we are already in a unrealistic housing market, I believe the best approach to assist the LGBTQIA+ community with housing is to build a new housing opportunity for the community.	0.07
85	Create LGBTQIA+ housing for community	0.07
90	Furnished affordable apartments	0
109	Shared housing	0
110	Permanent Supportive Housing	0
116	Affordable safe housing.	0.04
120	Inclusive housing, assisted living and long-term care for seniors	0
	Housing Types Cluster Statistics	
	Statement amount: 13	Average: 0.038
	Median: 0.036	Variance: 0.003
	Standard deviation: 0.051	Minimum: 0
	Maximum: 0.193	
